# The status of antioxidants, malondialdehyde and some trace elements in serum of patients with breast cancer

**Published:** 2016

**Authors:** Azadeh Sadati Zarrini, Dariush Moslemi, Hadi Parsian, Mahmood Vessal, Abbas Mosapour, Ziba Shirkhani Kelagari

**Affiliations:** 1 Department of Biology, Science and Research Branch, Islamic Azad University, Fars, Iran.; 2 Department of Radiation Oncology, Babol University of Medical Sciences, Babol, Iran.; 3 Cellular and Molecular Biology Research Center, Health Research Institute, Babol University of Medical Sciences, Babol, Iran.; 4 Department of Clinical Biochemistry, Babol University of Medical Sciences, Babol, Iran.; 5 Babol University of Medical Sciences, Babol, Iran.

**Keywords:** Antioxidant/oxidant status, Breast cancer, Antioxidant enzymes, Trace elements

## Abstract

**Background:**

There are studies that indicated dyshomeostasis of oxidant/antioxidant and trace elements in breast cancer patients, but the data regarding the status of these parameters in various stages of breast cancer are limited. The aim of this study was to highlight the status of these biochemical factors in various stages of breast cancer.

**Methods:**

Fifty-eight breast cancers patients participated in this study and underwent staging work up for the assessment of disease stage. Serum total antioxidant capacity and lipid peroxidation were determined spectrophotometically. Glutathione peroxidase (GPX), catalase (CAT) and superoxide dismutase (SOD) levels were analyzed by ELISA method. The serum level of Cu, Mn and Zn was measured by atomic absorption spectrophotometer. Student t-test and one-way analysis of variance (ANOVA) were used to compare group means.

**Results:**

All the patients included in the study classified as mild (stages I+II) and advanced stages (stages III+IV). Patients in advanced stage had lower serum antioxidant capacity and higher lipid peroxidation levels, but the differences were not statistically differet (P=0.690 and 0.666, respectively). Patients in advanced stage had higher, but not statistically different serum levels of CAT, GPX and SOD levels (p>0.05). Patients in both groups had to some extent similar serum Cu, Mn and Zn levels.

**Conclusion:**

There was no evidence of remarkable discrepancy in the status of analyzed factors in various stages of breast cancer. It seems that the severity of oxidative stress in different stages of breast cancer is similar to some extent.

Breast cancer is one of the main cancers that are most common among women. It was reported that each year, one million new patients are diagnosed with this fatal disease. In addition it is reported that death of 400,000 women annually is because of this disease. Several risk factors are introduced as causative factors that are able to involve the women. It is suggested that changes in inflammatory status, genetic alterations, changes in MicroRNA status because of exposure to ionizing radiation, oxidative stress and some trace elements are involved in the development of breast cancer (1-5). An imbalance between the various reactive oxygen and nitrogen species, accompanied with a decline antioxidant defense, is known as oxidative stress (5-7). When antioxidant scavengers are not able to scavenge the excess formed oxygen free radicals, oxidative stress will occur. Later, these radicals can damage important biomolecules such as DNA, lipids and proteins.

When peroxidation of these vital biomolecules occurs, various mutagen and carcinogen factors will produce (7, 8). Antioxidant defense system including enzymatic and non -enzymatic components which try to scavenge produced mutagen and carcinogen factors by free radicals that generated in cell (8-10). It is also reported that various trace elements can play role as carcinogenesis and interestingly some of them cooperate with antioxidant system for scavenging the free radicals. When cancer occurs, it is reasonable that imbalance in the status of oxidant/antioxidant system and trace element will occur. There are studies that indicated the dyshomeostasis of oxidant/antioxidant and also some trace elements in various cancers including breast cancer. But it is unclear that patients in higher stages of the disease had more dyshomeostasis in these parameters and the data regarding this matter is limited (11, 12). 

It is very important for both patients and physicians, highlighting the status of oxidant/antioxidant in various clinical and pathological stages of the disease. It seems that patients were more favor through intake of enriched foods in oxidant scavenger and also some of them, especially patients in advanced stages of cancer, consume antioxidant supplement during their treatment protocol. But what is important is this matter that do patients in higher stages of the disease should take more from these antioxidant enrich foods (13), and if is there any remarkable difference between the status of these parameters in early versus advanced stages of the disease. In this study, we aimed to compare the serum lipid peroxidation status, total antioxidant capacity and related enzymes such as catalase (CAT), superoxide dismutase (SOD), glutathione peroxidase (GPx), and trace elements such as zinc (Zn), copper (Cu) and manganese (Mn) in patients with various stages of breast cancer.

## Methods

**Study population:** The breast cancer patients were selected among patients who presented to Rajaei Hospital, Babolsar, Iran. Diagnosis of breast cancer was based on clinical and pathologic examinations. All patients underwent staging work up for the assessment of the stage of their cancer and evaluated by Tumor, Node and Metastasis (TNM) staging system (14). Demographic and clinical data of the patients including body mass index (BMI), age, status of estrogen and progesterone receptor, human epidermal growth factor receptor, histological grade and other related data were recorded for each patient. The treatment protocol for all patients was similar and all of them passed tumor resection surgery and chemotherapy. The chemotherapy regimen that used for patients were similar and include: Cytoxan 600mg/m^2^- Adriamycin 600mg/m^2 ^and 5-FU 600 mg/m2 (CAF) for six course at 3-weeks intervals. One month after the last day of the chemotherapy, 7 ml of blood samples were taken from each patient and immediately two milliliter of the sample was separated for CBC test. From the rest of the blood samples, serum was separated from *aliquot* in 0.2 ml micro tubes and stored at -80° C until final analysis.

**Analysis of total oxidant/antioxidant status:** Oxidative stress in cell can lead to the production of lipid hydroperoxides from polyunsaturated fatty acids that will form malondialdehyde (MDA), which can be quantified in reaction with thiobarbituric acid (TBA). Reaction of MDA with two TBA molecules and finally elimination of two water molecules leads to the formation of a chromogen that has maximum absorbance at 532nm. Measurement of the levels of MDA was considered as total oxidant status and known as thiobarbituric acid reactive substances (TBARS) test (15).

Ferric ion reducing antioxidant power (FRAP) test was used for the determination of the total antioxidant capacity. Simply, the principle of the test is the reduction of ferric ion to ferrous from in the presence of antioxidants at low PH, and determination of maximum absorbance of the colored ferrous-tripyridyl-S-triazine (Fe (III)-TPTZ) complex at 593nm by spectrophotometer (16).

**Measurement of serum trace element levels:** Serum copper (Cu), manganese (Mn) and zinc (Zn) levels were measured by atomic absorption spectrophotometery method (PG-990, china). Amounts of Zn and Cu were measured by flame method, while the serum level of Mn was measured using graphic furnace according to the instrument instruction. Serum for the measurement of Cu was diluted ten times with 0.1N HNO_3_. Various working standards i.e. 1.25, 0.625, 0.312, 0.156, 0.078, 0.039 and 0.019 PPM were used for preparing the standard curve from 1000 PPM stock of Cu (Merck, Germany). For the measurement of Zn, we used Zn stock (1000 PPM, Merck, Germany) and diluted the stock with 0.5 M nitric acid for preparing the different concentrations of Zn (0.625, 0.312, 0.156, 0.078, 0.039 and 0.009 PPM). All serum was diluted ten times with distilled water and concentration of Zn was determined in the diluted serum. To measure the levels of Mn in serum, all samples were diluted with distilled water (1:2 ratios). Standard of Mn solution (1000 PPb) was prepared from the KMnO_4_ powder (Merck, Germany) and various working standards were prepared (12.5, 6.25, 3.12, 1.78, 0.85 and 0.425 PPb) from the main stock with 0.1N HNO_3_. 

**Measurement of serum antioxidant enzymes:** For analyzing the levels of glutathione peroxidase (GPX), catalase (CAT) and superoxide dismutase (SOD), ELISA method was used. Amount of GPX was measured by GPX kit (CSB-E09496h, Cusabio Company, China) and expressed as μIU/ml, CAT enzyme was determined by CAT Kit (CSB-E13635h, Cusabio Company, China) and the results were reported as Pg/ml. Finally the amount of serum SOD was analyzed by SOD kit (CSB-EL022399HU, Cusabio Company, China) and the levels of the enzyme were reported as Pg/ml. These assays employ the quantitative sandwich enzyme immunoassay technique. Antibody specific for each enzyme has been pre-coated onto a microplate. Standards and samples are pipetted into the wells and any present enzyme is bound by the immobilized antibody. After adding biotin-conjugated antibody specific for each enzyme and avidin conjugated horseradish peroxidase, substrate solution is added to the wells and colors develop in proportion to the amount of enzymes bound in the initial step. In the final step, the color intensities were measured in appropriate wavelengths, on an ELISA reader (Awareness - America).

**Statistical analysis: **Because of the small number of the patients that were in stages I and IV, we considered stages I and II of the disease as mild stage and the stages of III and IV of the disease as advanced stage of the disease. Student t test was used for the analysis of the differences between the mean of variables (serum lipid peroxidation status, total antioxidant capacity, CAT, SOD, GPx, and trace elements) in two groups. One-way analysis of variance (ANOVA), following least significant difference (LSD) test was used for multiple comparisons. A p-value less than 0.05 was considered statistically significant. All statistical tests were two-sided and analyses were carried out using SPSS 18.0 (PASW, USA).

## Results

Fifty-eight patients were included in this study. Characteristics of patients including demographic and pathological data are presented in table 1. As it is clear from this table, most patients were in stages II and III of the disease. In addition, more than half of the patients were estrogen and progesterone receptor positive, while mostly included women that were Her-2 negative and 93.1% had invasive ductal carcinoma.

**Table1:** Characteristics of breast cancer patient included in this study

**Mean±SD of variable**	**Number (%)**
Age (years) 51.512.2	58 (100)
BMI( kg/m^2^) 29.3 5.0	58 (100)
**Clinical Stage **	
Stage I Stage II Stage III Stage IV	4 (6.9) 29 (50.0) 21 (36.2) 4 (6.9)
**Estrogen and Progesterone receptor**	
Positive Negative	38 (65.5) 20 (34.5)
**Her-2**	
Positive Negative	9 (15.5) 49 (84.5)
**Histology type**	
IDC ILC	54 (93.1) 4 (6.9)
**Lymph node involvement**	
N0 N1 N2 N3	16 (27.6) 20 (34.5) 16 (27.5) 6 (10.4)

Age and BMI are expressed as mean ± standard deviation (SD); BMI was calculated as weight/height^2^ (kg/m^2^); Her-2: human epidermal growth factor receptor; IDC: invasive ductal carcinoma; ILC: invasive lobular carcinoma

The results of hematological tests in four stages of the disease are presented in table 2. The results of CBC test were similar across the four study groups except WBC count and mean cell hemoglobin (MCH). In table 3, the results of multiple comparison of various parameters i.e. antioxidant/oxidan levels, CAT, GPX, SOD and trace elements within four stages of breast cancer by ANOVA, LSD test are presented. As this table shows, in patients with higher stages of the disease, the levels of oxidant and antioxidant were nonsignificantly higher than others groups. Patients in the early stages of the disease had higher levels of CAT, GPX and SOD (p>0.05). Differences in the levels of Cu, Mn and Zn also did not reach a significant level. We compared the results of hematological and biochemical parameters of mild and advanced groups and the results are presented in tables 4 and 5. Hematological variables in mild and advanced stages of the disease did not have statistical difference except for MCH levels. Furthermore, antioxidant/oxidan status, levels of CAT, GPX and SOD enzymes and also trace elements levels in two groups of mild and advanced were not statistically different.

**Table 2:** Comparison between hematological variables of patients in four stages of breast cancer by ANOVA

**Variable**	**Stage I**	**Stage II**	**Stage III**	**Stage IV**	**P-value**
WBC(µL^-1)^	3.22±0.89	5.29±1.30	5.63±1.93	6.30±1.50	0.030
RBC(µL^-1)^	4.10±0.21	3.93±0.43	3.94±0.38	4.30±0.62	0.277
HGB(g/dL)	11.5±0.3	10.9±1.1	10.5±0.9	10.7±1.7	0.360
HCT(g/dL)	35.9±0.5	34.5±2.9	33.8±2.3	38.2±6.3	0.051
MCV(fL)	87.1±5.6	88.3±4.9	86.1±5.8	88.1±9.7	0.607
MCH(pg)	28±1.9	27.8±1.3	26.7±2.2	24.9±4.5	0.039
MCHC(g/dL)	31.8±0.9	31.4±1.1	31±1.5	28.7±6.6	0.077
PLT(µL^-1^)	247.5±75	253.5±49.9	254.3±52.2	242.7±82.7	0.978
Lym%	35.5±9	30.4±7.7	30±9.5	27.5±5.2	0.580

Abbreviations; WBC: white blood cells, RBC: red blood cells, HGB: hemoglobin, HTC: hematocrit, MCV: mean cell volume, MCH: mean cell hemoglobin, MCHC: mean cell hemoglobin concentration, PLT: platelet, LYM: lymphocytes.

**Table 3:** Comparison between various antioxidant/oxidan status and trace elements in different stages of breast cancer by ANOVA. Data are expressed as Mean±SEM

**Variable **	**Stage I**	**Stage II**	**Stage III**	**Stage IV**	**P-value**
FRAP (µmol/L)	551.3±2.9	651.2±17.2	612.3±29.6	693.3±66.4	0.195
TBARS(µmol/L)	1.97±0.26	2.08±0.05	2.07±0.04	2.29±0.13	0.361
CAT (pg/ml)	1180.6±131.9	771.1±78.3	988.4±126.4	617.6±264.5	0.174
GPX (µIU/ml)	246.2± 90.0	93.5±18.1	127.9±34.5	43.6±8.4	0.098
SOD (pg/ml)	412.9±162.3	254.7±80.5	408.7±124.3	80.3±51.0	0.492
Cu (ppm)	1.5±0.25	1.2±0.05	1.3±0.07	1.3±0.30	0.459
Mn (ppb)	25.7±2.7	24.3±3.5	30.1±2.9	31.4±12.2	0.633

FRAP: ferric reducing antioxidant power, TBARS: thiobarbituric acid reactive substances, CAT: catalase, SOD: superoxide dismutase, GPX: glutathione peroxidase

**Table 4:** Comparison between demographic and hematological variables in mild and advanced stages of breast cancer

**Variable**	**StageI+II (n=33)**	**Stage III+IV (n=25)**	**P-value**
WBC(µL^-1)^	5.05±1.43	5.74±1.86	0.113
RBC(µL^-1)^	3.95±0.41	4.01±0.44	0.599
HGB(g/dL)	10.98±1.09	10.56±1.02	0.137
HCT(g/dL)	34.75±2.76	34.56±3.49	0.814
MCV(fL)	88.29±4.90	86.50±6.39	0.232
MCH(pg)	27.84±1.42	26.49±2.73	0.030
MCHC(g/dL)	31.53±1.12	30.70±2.88	0.135
PLT(µL^-1^)	252.85±52.10	252.48±56.11	0.980
Lym%	31.07±7.97	29.66±8.93	0.529
Age (years)	51.3±12.2	51.8±12.3	0.886
BMI( kg/m^2^)	30.0±5.1	28.3±4.8	0.199

**Table 5:** Comparison between various antioxidant/oxidan status and trace elements in two groups of breast cancer (mild and advanced stages) by ANOVA

**Variable**	**Stage I+II (n=33)**	**Stage III+IV (n=25)**	**P-value**
FRAP (µmol/L)	638.7±17.2	626.3±27.2	0.690
TBARS(µmol/L)	2.0±0.05	2.1±0.04	0.666
CAT (pg/ml)	822.3±74.0	929.0±115.6	0.422
GPX (µIU/ml)	112.0±20.5	114.4±29.5	0.946
SOD (pg/ml)	273.8±73.2	356.1±107.0	0.515
Cu (ppm)	1.23±0.05	1.28±0.07	0.645
Mn (ppb)	24.5±3.0	30.3±3.1	0.191
Zn (ppm)	0.75±0.03	0.69±0.03	0.250

## Discussion

In this study, the serum levels of total oxidant/antioxidant and trace element in various stages of breast cancer were compared. Patients participated in the study one month after the chemotherapy intervention. It seems that the effect of chemotherapy drugs disappeared, because most of the hematological parameters were in normal range. Although some discrepancies in the status of serum total oxidant/antioxidant, related enzymes and trace element levels in various stages of breast cancer were observed, but the differences were not statistically significant. 

There are several studies that determined the status of oxidant/antioxidant in breast cancer patients. In a study by Sheeba et al. on breast cancer patients, they showed that patients had higher concentration of MDA than the normal ones (8). Gonenc et al. showed that the differences in the levels of serum MDA in stage II, III and IV in comparison of control group was significant (10). We also observe such a pattern and patients in stage IV had the higher levels of MDA in their serum. The increase of MDA showed the increasing oxidative condition in mild stage toward advanced stages. In this condition for compensation, body tries to increase antioxidant system levels (see table 3, FRAP test). In another study, serum MDA levels decreased in breast cancer patients in comparison to benign group (p<0.05) (17). Yeh et al. reported an increase in the rate of MDA, activities of SOD, GPx and GPx in breast cancer patients vs. normal ones (18). Ray et al. observed that MDA concentration had significant increase in stage II and III of the disease in comparison to control. SOD and GPx, but not CAT activities were significantly increased in patients (19). We observed that the levels of these enzymes decreased from stage I toward stage IV. 

Kuo et al. determined levels of Zn, Cu, Selenium (Se) and iron in the serum and tissue of breast benign and malignant patients. They observed higher levels of Se and Zn in control than the other two groups. Blood Fe and Cu levels in malignant group were higher than normal and benign groups (11). Siddiqui et al. determined the status of Pb, Zn, Cu, Fe and Ca on benign and malignant breast cancer and found that the levels of these elements were significantly higher in malignant cases than benign ones (12).

Mn and Cu are important co-factors of antioxidant enzymes such as superoxide dismutase (20, 21). We observed the value of Cu and Mn increased in serum in accordance with severity of the disease. It seems that in the first steps of cancer, when the rate of active species of oxygen and free radicals increase slightly, body tries to increase the levels of this enzyme. SOD will enlist serum Cu and Mn and ultimately may the rate of Cu and Mn decreases. But by increasing the intensity of disease and the amount of free radicals, the rate of enzymes decreases. Therefore, more values of Mn and Cu are seen in the serum of patients. Zinc is also the co-factor of superoxide dismutase (20, 21) and it seems that when dyshomeostasis occurs, the hemostasis of this cofactor is disturbed, as we have seen in our study. 

In conclusion, this study showed that there was no evidence of remarkable discrepancy in the status of antioxidant/oxidant, related enzymes and trace elements in various stages of breast cancer. It seems that the severity of oxidative stress in different stages of breast cancer is similar to some extent. If we were able to compare the levels of analyzed variable with healthy control group, perhaps we would be able to find a significant difference. 

Because of the small sample size and lack of healthy control group for more precise comparison, this study is not conclusive. While we tried to select the patients with similar chemotherapy regimen, this intervention can affect the results. Definitely, a study with bigger sample size along with control group and analyzing the status of the abovementioned parameters in another study are needed.

## Notes

**Funding: **The authors are grateful to Babol University of Medical Sciences for their support.
